# Role of Lifestyle in Thoracic Outlet Syndrome: A Narrative Review

**DOI:** 10.3390/jcm13020417

**Published:** 2024-01-11

**Authors:** Pierre Abraham, Simon Lecoq, Muriel Mechenin, Eva Deveze, Jeanne Hersant, Samir Henni

**Affiliations:** 1 Service of Sports Medicine, University Hospital, 49100 Angers, France; simon.lecoq@chu-angers.fr; 2 Service of Vascular Medicine, University Hospital, 49100 Angers, Francejeanne.hersant@chu-angers.fr (J.H.);; 3 INSERM, CNRS, MITOVASC, Equipe CarMe, SFR ICAT, University Angers, 49100 Angers, France; 4 Service of Thoracic and Vascular Surgery, University Hospital, 49100 Angers, France

**Keywords:** movement, pain, upper limb, exercise, neurovascular compression

## Abstract

Introduction: The presence of a positional compression of the neurovascular bundle in the outlet between the thorax and the upper limb during arm movements (mainly abduction) is common but remains asymptomatic in most adults. Nevertheless, a certain number of subjects with thoracic outlet positional compression will develop incapacitating symptoms or clinical complications as a result of this condition. Symptomatic forms of positional neurovascular bundle compression are referred to as “thoracic outlet syndrome” (TOS). Materials and methods: This paper aims to review the literature and discuss the interactions between aspects of patients’ lifestyles in TOS. The manuscript will be organized to report (1) the historical importance of lifestyle evolution on TOS; (2) the evaluation of lifestyle in the clinical routine of TOS-suspected patients, with a description of both the methods for lifestyle evaluation in the clinical routine and the role of lifestyle in the occurrence and characteristics of TOS; and (3) the influence of lifestyle on the treatment options of TOS, with a description of both the treatment of TOS through lifestyle changes and the influence of lifestyle on the invasive treatment options of TOS. Results: We report that in patients with TOS, lifestyle (1) is closely related to anatomical changes with human evolution; (2) is poorly evaluated by questionnaires and is one of the factors that may induce symptoms; (3) influences the sex ratio in symptomatic athletes and likely explains why so many people with positional compression remain asymptomatic; and (4) can sometimes be modified to improve symptoms and potentially alter the range of interventional treatment options available. Conclusions: Detailed descriptions of the lifestyles of patients with suspected TOS should be carefully analysed and reported.

## 1. Introduction

The anatomy of the shoulder predisposes the neurovascular bundle towards the upper limb to compressions during abduction of the arm at different levels of the thoracic outlet pathway [1,2,3]. This neurovascular positional compression may result in pain, discomfort, or complications and, in such cases, is referred to as thoracic outlet syndrome (TOS). TOS is classified as a rare disease on both sides of the Atlantic (“NORD^®^” rare disease database/ORPHANET n°97330), with the incidence of TOS estimated to be between 2.5 and 4.0 cases per 100,000 people per year in the United States [4,5], and 3 to 4 cases per 100,000 people per year in France [6]. The high frequency of observations in athletes [7,8,9] has led some authors to conclude that the prevalence of TOS may be much higher in these subjects [1].

The clinical issue is that screening for positional compression in the general population results in a prevalence of positive tests much higher than the prevalence of TOS cases [10,11,12,13]. Indeed, positional compression of the neurovascular bundle remains completely asymptomatic in most subjects. To date, asymptomatic cases of neurovascular positional compression have led many authors to suggest that tests used to diagnose TOS either perform poorly or have little discriminative value [11,14,15,16,17]. We recently proposed that these cases of positional compression without symptoms or complications should not be named TOS but “thoracic outlet compressions” (TOCs) in order to avoid confusion between the two conditions [18]. Ultimately, a small number of patients with TOC or TOS will develop complications or sequelae (CS) as a result of the chronic conflict between the neurovascular bundle and the adjacent structures, such as vascular thrombus or aneurysms [19,20,21], Gilliatt and Sumner hand, and peri-neural fibrosis [22,23,24,25,26]. We also proposed that such cases of “thoracic outlet compression with complications or sequelae” should not be called TOS because the complication may be asymptomatic; instead, they should be referred to as “TOX” (which is a simplified version of TOC-CS) [18]. This TOC-TOS-TOX paradigm will be used throughout the present manuscript [18].

When TOS occurs as a result of the compression of nerves or vessels by adjacent structures during upper-limb movements, a large variety of signs and symptoms can be present [5,27]. These symptoms are generally transient during upper-limb movements or activities, but complications may also occur at the venous level (Paget–Schroetter Syndrome) [28,29,30], at the arterial level (sub-clavian aneurysm or distal emboli) [31,32,33], or at the neural level (Gilliatt and Sumner syndrome) [26,34].

Since it is widely admitted that TOS should be classified as neural TOS (n-TOS) in cases in which no arterial or venous complication is observed, n-TOS would represent 90% of cases, occurring mainly in females, venous TOS (v-TOS) would represent 10% of cases with male dominance, and arterial TOS (a-TOS) is considered sporadic.

In brief, there is an enormous difference between the incidence of patients diagnosed with TOS or TOX and the prevalence of TOC in the population. There are also differences in the populations that are affected by n-TOS or by v-TOS. We believe that lifestyle might be an essential determinant of these differences. We therefore aimed to review lifestyle issues in cases of TOS and TOX, arguing that TOS and lifestyle are inextricably linked. We divided our study into sub-chapters, discussing (1) the historical importance of lifestyle (and anatomy) evolution in TOS; (2) the methods for lifestyle evaluation in the clinical routine of TOS-suspected patients; (3) the role of lifestyle in the occurrence and characteristics of TOS or TOX; and (4) the influence of lifestyle on the treatment options for TOS and TOX, respectively.

## 2. Historical Importance of Lifestyle Evolution on TOC and TOS

Evolutionary changes from our simian ancestors have allowed or accompanied a simultaneous evolution of motor and cognitive functions (and thereby of lifestyle) in humans but have also resulted in profound changes in the morphological characteristics of the shoulder and of the clavicle [35,36]. The point here is not to discuss whether lifestyle changes have induced anatomical changes from hominid ancestors or if changes in anatomy have enabled lifestyle changes. Essentially, compared to apes, the human clavicular curvature reduces the costo-clavicular angle [36]. The anatomy of the modern human predisposes the neurovascular bundle to TOC at different levels of the thoracic outlet, specifically during abduction or elevation of the arm [27]. The morphology of the clavicular bone in individual evolution also changes with age and gender [37,38]. The male clavicle is longer, wider, and thicker than in a human female [39], which is partly explained by the difference in stature [40] and may be a possible explanation of the higher occurrence of TOS in females than in males. Curvature itself increases not only in childhood but also during adulthood [37,38]. Ethnic differences may also exist with a significant relationship between the height of the subjects and the length of their clavicle, with a comparable slope but longer clavicles in Asian people than in Caucasians [40].

The cervical rib is also known to be a risk factor for TOS. Primates, including the great apes [41], have on average 13 pairs of ribs, while humans only have 12 pairs. The presence of a cervical rib is a relatively rare anatomical trait, affecting 0 to 3.4% of the modern population [42,43]. It is thought to have been described by Claudius Galenus in the first century and was found on skeletons from the classic Kerma period in the Nile valley (approx. 1600 BC) [42]. Whether the presence of cervical ribs in modern humans relates to persistent simian traits or results from genetic specificities of the *Hox* genes and growth differentiation factor 11 [44] is still open to debate [45]. Based on systematic CT scan analysis, research has proposed that female patients are more likely to have cervical ribs than male patients, while the elongated C7 transverse process may be more frequent in males [43], with possible ethnic variability [46].

## 3. Methods for Lifestyle Evaluation in the Clinical Routine of TOS-Suspected Patients

If determining lifestyle is important in managing and treating TOS, it is important that it be carried out in a clinical routine setting. Lifestyle in TOS patients is, however, likely largely under-evaluated. Many tools are used or proposed in TOS populations, such as the “short form” SF12 or SF36, the “Functional Evaluation in Thoracic Outlet Syndrome” (FETOS) [47], or the “Thoracic outlet syndrome index” (TOSI) [48]. Table 1 reports the list of tasks proposed for some of the available tools. The questionnaires recommended by the society for vascular surgery in TOS evaluation include the Disabilities of the Arm, Shoulder, and Hand (DASH) questionnaire and its shortened version (quick-DASH) and the Cervico-Brachial Symptom Questionnaire (CBSQ) [27,49].

The DASH evaluates the degree of difficulty involved in performing various tasks (21 items whose responses range from “no difficulty” to “unable”). It also evaluates the effects on social activities, work or sleep (three items), the severity of pain (five items), the impact on self-image, and optional modules regarding the degree of difficulty of work and sports/performing arts (four items) [27]. The quick-DASH is a shortened 11-item version of the DASH and has been used in a number of recent studies of TOS patients. Nevertheless, it is of interest to note that, except for washing one’s back, none of the tasks requiring significant arm abduction or elevation that are present in the DASH are included in the quick-DASH version. The CBSQ is also poor in the evaluation of lifestyle since only one item (item 3) includes overhead activities [27]. The TOSI evaluates (on a 10-point visual analogue scale) how much pain or weakness is experienced and how much the disease affects sports, work, or lifestyle [48]. The FETOS self-questionnaire comprises 16 items rated on a four-point scale: impossible; major discomfort; moderate discomfort; or no difficulty [47]. Nevertheless, none of these questionnaires evaluate whether each task is or is not performed or, if performed, how often it occurs each day or week.

There are a number of lifestyle questionnaires that focus on the frequency by which selected tasks are performed, including the healthy lifestyle questionnaire for the elderly (Heal), but to the best of our knowledge, these have never been used in the context of TOS, and of the total 35 items of the Heal, only 3 relate to everyday tasks while 2 items relate to sports [50]. Similarly, only 2 of the 25 items of the “Fantastic lifestyle assessment” tool relate to activity, one of which is not specific while the other concerns the lower limbs [51]. The only thing that is frequently reported in TOS series and case reports is the profession of the patient when the study deals with work-induced upper-limb disease and the types of sport activities pursued by athletes [52,53,54,55,56,57,58,59,60,61,62,63].

We believe that it is important to ask about the difficulty involved in performing tasks (such as those included in the DASH questionnaire). Nevertheless, asking whether a task was performed is essential, and where not, it is crucial to know why it has not been performed. Indeed, if the patient rarely faces the task in question in everyday life, it is unlikely that the difficulty rating will be high. For example, it seems irrelevant to ask someone living in a flat that has no garden to evaluate the level of difficulty involved in gardening or doing outdoor work (as in the DASH questionnaire), to ask an unemployed person how much TOS disturbs their job (as in the TOSI questionnaire), or to ask a vegan person to evaluate the level of difficulty involved in carving a roast (as in the FETOS questionnaire). At the same time, however, it may well be the case that the patient has developed an avoidance [64] behaviour. For pain-related fear and avoidance, tools for patients with lower-back pain or pain not specific to the upper limb can be proposed for upper-limb evaluation [65,66]. A specific tool to estimate the avoidance of daily activities of the shoulder has recently been proposed [67]. Nevertheless, to the best of our knowledge, this latter tool has never been tested in the context of a population with suspected TOS.

## 4. Role of Lifestyle in the Occurrence and Characteristics of TOS

Beyond eventual trauma to the shoulder, it is generally acknowledged that the increase in muscle size due to chronic activity or training reduces the spaces in the thoracic outlet pathways and increases the risk and severity of compression of the neurovascular bundle [68]. This is the most likely explanation for the fact that TOS is more frequent among physically active workers who use their arms [69,70] than among white-collar workers [71]. Similarly, in athletes, TOS is particularly frequent in sports involving overhead activities that use the upper limb [7], although a recent review highlighted that TOS can also be observed in other sports (football, cycling, running, etc.) [8]. Among possible explanations is the possibility that TOS occurs if the frequency or duration of compression, and hence its consequences on neurovascular function, exceeds the level of post-compression recovery, explaining its particular frequency in certain professions that are characterized by repetitive movements of the upper limb. It is worth noting that among athletes, TOS is more frequent in males than in females—and frequently the venous type (Paget–Schroetter Syndrome) [7]—whereas neural TOS represents 90% of cases with a female dominance in most TOS series of the general population [4].

As previously underlined, TOS is “more prevalent in those with the shoulder flexed for the majority of the working day, have repeated trauma to the shoulder joint, and with those who exhibit abnormal posture, including positions required to play bowed instruments. Repeated trauma to the head or neck, postural dysfunction, extended duration in compromising shoulder positions, pregnancy, edema, anatomical deviations, hypertrophied muscles (e.g., scalenes), boney growths, and muscle weakness are all theorized to be contributing factors to TOS” [72].

These observations can also provide interesting directions that explain why most TOCs remain asymptomatic. On the one hand, the number and duration of movements inducing a compression in these patients are perhaps too small to result in symptoms. On the other hand, when questioning patients, it is a frequent observation that many apparently asymptomatic individuals develop avoidance behaviours since they have experienced difficulties in their daily routine (such as using a headset or earbuds to listen to their phone’s audio rather than holding it in their hand [73], bending their head to brush their hair, or holding a hand rail on public transport at hip level rather than overhead). As a consequence, such individuals no longer report complaints once their avoidance techniques have become fully integrated into their behaviour.

Another area of relatively recent interest is the occurrence of TOS in patients undergoing dialysis [74,75,76] or with pacemakers [77,78,79]. In both cases, the intervention seems to be able to induce symptoms in patients who had asymptomatic TOCs [76,77], and the presence of TOCs can induce complications on the intravascular leads [79].

## 5. Influence of Lifestyle on the Treatment Options of TOS

Most symptomatic patients with TOS are reported to benefit from physical therapy [80,81] or medical treatment [9,82,83]. Physical therapy leads to the resolution of symptoms, despite the persistence of TOC in many of these patients. Indeed, the majority of patients whose symptoms have improved will never require surgery. Surgery is generally only proposed when physical therapy, drug treatment, and/or lifestyle changes have failed to improve the symptoms [49].

### 5.1. Treating TOS through Lifestyle Changes

Although upper-limb exercise is a risk factor for TOS occurrence, as previously outlined, it could appear paradoxical that training has been shown to reduce neck and upper-limb symptoms [81,84,85]. Many previous studies have not specifically focused on TOS, but it is possible that the risk of symptoms in patients with TOC follows a J-shape relationship with physical activity. Furthermore, Levine and Rigby have extensively reviewed the biomechanical importance of tendinous and muscle structure on the static and dynamic stability of the shoulder in TOS and their consequences in terms of exercise as a treatment of TOS [72]. The issue is therefore not exercise as a whole but about encouraging specific activities or avoiding those that induce a compression. As showed by Li et al., positive prognostic factors that make the non-surgical treatment of TOS available “include physical therapy compliance, long-lasting postural and habitual lifestyle modifications, and a sedentary job with few physical demands” [86]. Beyond these, favouring specific physical activities to reinforce shoulder stability and modifying breathing patterns or head or pelvis alignment (by adapting or influencing body position during domestic or professional activities) are essential in treating TOS [72,81,87,88,89,90]. At the upper-limb level, the cost-effectiveness of intervening in the physical activities undertaken as part of one’s everyday lifestyle, in addition to intervening in working practices, on the recovery from neck and upper-limb symptoms in computer workers or performing artists has been reported [91,92], but little evidence is available regarding TOS exclusively. One of the reasons for this lack of evidence is the difficulty in providing objective proof of the relationship between symptoms and the presence of a positional compression. Recently developed approaches using STIR-MRI [93] or positional ENMG for neural TOS [94], transcutaneous oximetry for arterial TOS [95], or venous photoplethysmography for venous TOS [96] are future options for attributing the symptoms to TOC. These measures will hopefully provide more robust evidence for the responsibility of the compression on symptoms.

### 5.2. Influence of Lifestyle on the Invasive Treatment Options for TOS

Another influence of lifestyle on TOS treatment is its influence on treatment options for surgery (and/or fibrinolysis in patients with Paget–Schroetter Syndrome). On the one hand, it has been suggested that patients with severe symptoms who are not willing to alter their daily activities are probably better off undergoing surgical management [97] than older sedentary patients or patients for whom lifestyle or professional changes may lead to the improvement or resolution of symptoms. Thrombolysis is often proposed in the treatment of thrombosis [30] and is associated with improved vein patency and functional outcomes following the first rib resection in patients with Paget–Schroetter Syndrome [98]. Although catheter-directed thrombolysis is expected to reduce the risk of a haemorrhage in thrombolysis, some adverse effects may still be observed [28,99]. Surprisingly, studies that compare treatment options for upper-extremity deep vein thrombosis are generally performed based on the physician’s point of view or experience [100] or on the sole global risk-to-benefit ratio of treatments [101]. Nevertheless, studies rarely report willingness and demand on the part of patients [97]. It is likely that, where surgery is concerned, patients forced to perform repeated movements with their upper limbs for sports or professional activities will be more willing to accept the risk of complications from thrombolysis in cases of Paget–Schroetter Syndrome. Some authors also suggest that patients who have been treated surgically are more likely to return to their original lifestyle and sports activities than individuals who have not undergone surgical treatment [97]. Other factors, such as the degree of reimbursement and annual household income, may also be potential factors that account for an individual’s willingness to pay for surgical treatments [102]. A number of studies compare warfarin with oral anticoagulants at the lower-limb level [103], but a comparable analysis of fibrinolysis versus anticoagulation has not been evaluated in TOS. That said, beyond Paget–Schroetter Syndrome, although many authors still consider surgery the gold standard [104], despite the risks associated with surgery [105,106], non-surgical treatment options (physical therapy, botulinum toxin injection, ultrasound-guided hydrodissection) have been proven to be effective in treating TOS [82,83,107] and are of interest in reducing the period following surgery during which sporting activities are discouraged.

### 5.3. Conclusion and Directions for the Future

The interactions between lifestyle, thoracic outlet compression, and thoracic outlet syndrome, with or without complications, are of major interest. Lifestyle changes from our simian ancestors are closely related to anatomical changes during human evolution, although the causality of bipedal walking over changes in shoulder anatomy cannot be proven. Lifestyle is one of the factors that may induce symptoms in patients with positional-induced neurovascular bundle compression. The absence of being exposed to manoeuvres that induce compression is one possible explanation for the fact that most patients with TOCs remain asymptomatic. As such, lifestyle is also an essential determinant in treatment choices, among which lifestyle changes (when possible) can be effective in treating symptomatic patients. The clinical determination and reporting of lifestyle in patients with suspected TOS should be systematic. Lifestyle remains poorly evaluated. Most questionnaires to date focus on the degree of difficulty or level to which patients avoid performing various tasks, but they do not determine whether and, if so, how often the subjects are exposed to these tasks. An analysis of exposure should be performed in the future when estimating the changes in upper-limb disability scores resulting from treatments to confirm that disability improvement does not only result from a decreased exposure level.

## Figures and Tables

**Table 1:** List of tasks from four questionnaires proposed in the TOS evaluation: Note that for the DASH, *X* indicates the items used in the quick-DASH version of the questionnaire.

DASH Questionnaire * 1. * Open a tight or new jar 2. Write 3. Turn a key 4. Prepare a meal 5. Push open a heavy door 6. Place an object on a shelf above your head *7*. Do heavy household chores (e.g., wash walls, wash floors) 8. Garden or do outdoor work 9. Make a bed *10.*. Carry a shopping bag or briefcase 11. Carry a heavy object (over 10 lb) 12. Change a lightbulb overhead 13. Wash or blow dry your hair *14.* Wash your back 15. Put on a pullover *16.* Use a knife to cut food *17.* Recreational activities that require little effort (e.g., cardplaying, knitting, etc.) 18. Recreational activities that require some force or impact (e.g., golf, hammering, tennis, etc.) 19. Recreational activities that require you to move your arm freely (e.g., playing frisbee, badminton, etc.) 20. Managing transportation needs (getting from one place to another) 21. Sexual activities *22.* Interference with normal social activities with family, friends, neighbours, or groups *23.* Limitation in work or other regular daily activities or any specific activity *24.* Symptoms of pain 25. Symptoms of pain during specific activities *26.* Symptoms of a tingling sensation 27. Symptoms of weakness 28. Symptoms of stiffness *29.* Difficulty sleeping 30. Impact on self-image CBSQ Questionnaire 1. Pain due to neck movement 2. Pain due to brief shoulder movement 3. Pain or fatigue with arm overhead 4. Swelling after arm exercise 5. Sensation of tingling or numbness when moving arm 6. Sensation of tingling or numbness when awakening from sleep 7. Sensation of tingling or numbness when writing 8. Sensation of tingling or numbness when grasping 9. Sensation of tingling or numbness when leaning on elbow 10. Clumsiness when trying to hold objects 11. Pain due to experiences that are normally not painful 12. Disabling long-lasting pain after light activities TOSI Questionnaire Pain and physical symptoms Pain in the shoulder and upper extremities Pain experienced in your axilla, thorax, neck, or cheek Weakness in the upper extremities Numbness/tingling sensation in the upper extremities Soreness and tiredness when using the hand/upper extremity, especially overhead Numbness and tingling sensation when waking up Weakness or clumsiness while trying to hold onto objects or while attempting to open jars, use a key to open a lock, pull a zipper or button clothing Sports and recreation activities 8. in your daily activities relating to house or yard work 9. Disturbance in your recreational activities Work 10. Difficulties experienced when working above head height 11. Time spent using the uninvolved arm to compensate for the injured one 12. Disturbance in your work in your job 13. Disturbance in your heavy household chores (washing windows, spring cleaning) Lifestyle 14. Disturbance in your sleep Emotions 15. Level of concern about the effect of TOS on your occupation or work FETOS Questionnaire Carry a shopping bag Push a caddy Carry a heavy object Peel vegetables Carve a roast Carry a full pan Reach a high-level shelf Screw or unscrew Sweep Iron Wash windows Brush your hair Wash yourself Carry out a professional activity Drive a car Have a good night’s sleep

## Data Availability

No new data were created or analyzed in this study. Data sharing is not applicable to this article.
